# The Effect of Simulated Dose Reduction on the Performance of Artificial Intelligence in Chest Radiography

**DOI:** 10.3390/jimaging11030090

**Published:** 2025-03-19

**Authors:** Hendrik Erenstein, Wim P. Krijnen, Annemieke van der Heij-Meijer, Peter van Ooijen

**Affiliations:** 1 Department of Medical Imaging and Radiation Therapy, Hanze University of Applied Sciences, 9714 CA Groningen, The Netherlands; a.van.der.heij-meijer@pl.hanze.nl; 2 Department of Radiotherapy, University of Groningen, University Medical Centre Groningen, 9713 GZ Groningen, The Netherlands; p.m.a.van.ooijen@umcg.nl; 3 Research Group Healthy Ageing, Allied Health Care and Nursing, Hanze University of Applied Sciences, 9714 CA Groningen, The Netherlands; w.p.krijnen@pl.hanze.nl; 4 Bernoulli Institute for Mathematics, Computer Science and Artificial Intelligence, University of Groningen, 9747 AG Groningen, The Netherlands; 5 Data Science Center in Health, University Medical Centre Groningen, 9713 GZ Groningen, The Netherlands

**Keywords:** AI, dose reduction, noise, image quality, chest radiography

## Abstract

Chest imaging plays a pivotal role in screening and monitoring patients, and various predictive artificial intelligence (AI) models have been developed in support of this. However, little is known about the effect of decreasing the radiation dose and, thus, image quality on AI performance. This study aims to design a low-dose simulation and evaluate the effect of this simulation on the performance of CNNs in plain chest radiography. Seven pathology labels and corresponding images from Medical Information Mart for Intensive Care datasets were used to train AI models at two spatial resolutions. These 14 models were tested using the original images, 50% and 75% low-dose simulations. We compared the area under the receiver operator characteristic (AUROC) of the original images and both simulations using DeLong testing. The average absolute change in AUROC related to simulated dose reduction for both resolutions was <0.005, and none exceeded a change of 0.014. Of the 28 test sets, 6 were significantly different. An assessment of predictions, performed through the splitting of the data by gender and patient positioning, showed a similar trend. The effect of simulated dose reductions on CNN performance, although significant in 6 of 28 cases, has minimal clinical impact. The effect of patient positioning exceeds that of dose reduction.

## 1. Introduction

Plain chest radiography is one of the most common radiological examinations. It plays a pivotal role in patient management and screening due to its accessibility and broad range of indications [1,2]. The diagnostic accuracy of screening by radiologists is directly impacted by image quality [3,4,5]. Image quality, in turn, is limited by radiation exposure. By decreasing the radiation dose, noise will increase, which will reduce the distinctive capacity of images and, thus, overall image quality [3,4,5,6,7,8]. While image noise can be reduced by increasing the dose, this is constrained by the linear relationship between the carcinogenic nature and radiation exposure [9].

Noise reduction and, thus, an increase in image quality, can also be achieved by post-processing with noise reduction techniques [10]. These techniques enable increased image quality while implementing an ‘as low as reasonably achievable’ radiation exposure, referred to as the ALARA principle [11,12]. It is important to note that although these techniques reduce noise, they can also reduce the perceptibility of detail and thereby hinder diagnostic accuracy [13]. Hence, the implementation of post-processing should be carefully considered.

More recently, artificial intelligence (AI) has led to the development of more complex image techniques aimed at noise reduction [5,14,15,16,17]. An example of the application of AI-based noise reduction is computed tomography (CT), which can achieve significantly lower doses while maintaining diagnostic accuracy for pediatric patients, a group that is especially sensitive to radiation [16]. Likewise, the implementation of AI-based noise reduction has been shown to lead to increased lung nodule detection in CT imaging [15]. It is important to note that these AI techniques strive to improve image quality for the diagnostic accuracy of human observers.

In addition to image optimization, AI models have been increasingly implemented as a diagnostic tool in radiology [18,19]. Chest imaging has been a recurring application for the development of these AI models [20,21,22,23,24,25]. Initially, the clinical representativity of popular datasets used for the development of AI models was limited (e.g., low resolution in the popular ChestX-ray14 introduced in 2017) [26,27]. More recently, high-quality data have been released, such as Medical Information Mart for Intensive Care—CXR (MIMIC-CXR), a dataset including full bit-depth images with clinically representative resolution [28,29,30,31,32]. AI models trained on either dataset tend to perform at a level comparable to radiologists in well-defined tasks, as shown by an area under the receiver operator curve (AUROC) > 0.90 [20,21,22,23,24,25]. Models trained on datasets of varying quality achieve comparable performance. Therefore, image resolution in plain chest radiology may have a limited impact on the training of AI models.

This observation was substantiated in a study by Sabottke et al. (2021), which showed that AI models maintain performance between resolutions of 600^2^ pixels and 128^2^ pixels, both of which are lower resolutions compared to those used in clinical practice (>1024^2^ pixels) [33]. The sustained performance is likely attributed to the use of convolutions and down-scaling of resolution, as is implemented by convolutional neural networks (CNNs), a commonly used model architecture. As noise in chest imaging is expressed at high resolutions, we hypothesize that the impact of reduction-induced noise on CNN performance differs from that of human observers. To the best of our knowledge, applying dose reductions to assess the impact of noise on CNN performance in plain chest imaging has not yet been investigated.

Assessing the impact of noise due to dose reduction on CNN performance of plain chest radiology is preferably performed using a set of images that differ in dose levels. However, this requires multiple exposures per case, which is ethically not feasible due to the aforementioned carcinogenic nature of radiation exposure. One way to circumvent the challenge of varying exposure per radiograph is to simulate low doses for an individual image, an approach previously applied in computed tomography [34,35].

The aim of this experimental study is to design a novel low-dose simulation and to evaluate the effect of this low-dose simulation on the performance of CNNs in plain chest radiography.

## 2. Materials and Methods

Two datasets were used in this study. The first dataset consisted of plain chest phantom images acquired for this study to develop a low-dose simulation (see Section 2.1). The second dataset, MIMIC, provided by the Massachusetts Institute of Technology (MIT), was used for CNN training, validation, and testing (see Section 2.2) [28,29,36,37].

**Figure 1:**
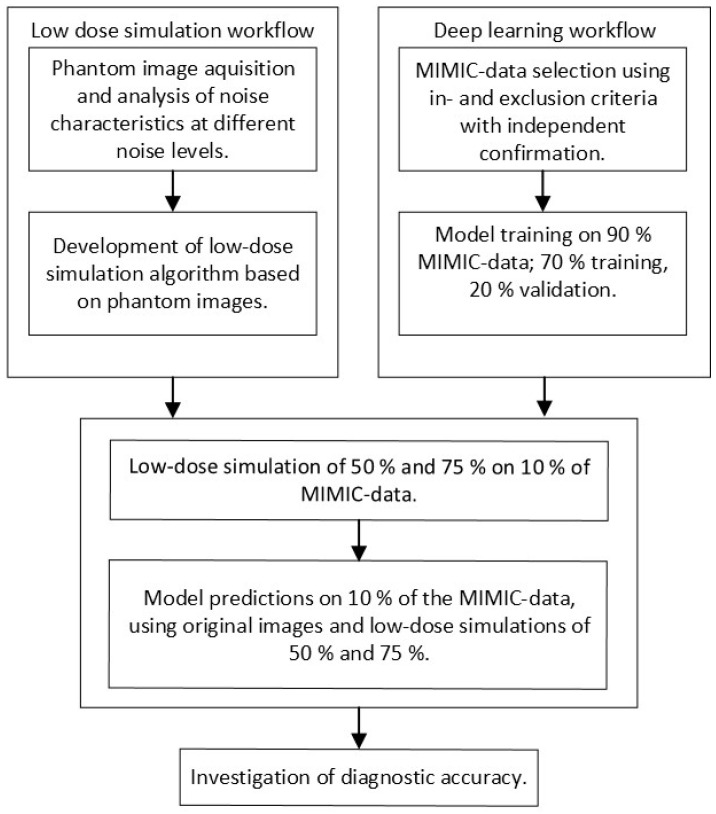
Visualization of the low-dose simulation, deep learning, and testing workflows used in this study.

This study was performed in four steps, which are schematically represented in Figure 1. First, a low-dose imaging simulation procedure was developed based on X-ray images obtained for this study using an anthropomorphic phantom. This simulation made it possible to process a single examination at different dose levels. Second, the MIMIC dataset, containing original chest radiographs, was used to train CNNs. Third, the developed low-dose simulation was applied to 10% of the MIMIC radiographs for independent testing. Fourth, the effect of simulated dose reduction on CNN performance was investigated. During the investigation, subpopulations were considered by stratifying for gender (male and female) and positioning (posterior–anterior (PA) and anterior–posterior (AP)).

### 2.1. Simulated Low-Dose Imaging

To analyze the relationship between noise and exposure parameters, a set of chest images was acquired using an anthropomorphic Alderson phantom containing tissue-equivalent materials representing different organs. A common range of clinically relevant exposure parameters for chest radiography was used with tube voltages (80, 100, and 120 kVp) and manual exposure time products (0.6, 0.8, 1.2, 1.6, and 2 mAs), resulting in 15 separate images. Note that the tube voltages within MIMIC have a mean (SD) of 101 (±12) kVp and fall within this range. All images were acquired at a source-to-image distance (SID) of 150 cm. This approximates the distances used in the MIMIC dataset, which are 183 (±0.6) cm and 165 (±3.2) cm for PA and AP images, respectively. The resulting phantom images were analyzed using 10 × 10-pixel sliding windows to measure the mean and standard deviation of the grey values, representing an approximation of noise. Linear regression was used to approximate the relationship between the mean grey values (horizontal axis) and standard deviation (vertical axis) for each tube voltage and exposure time product combination. To overcome systematic error in direct exposures, where grey values are 0 and the standard deviation is negligible, the *x*- and *y*-axis intercepts were set to 0. The resulting regression slope represents the variation in SNR per anatomical region caused by a variation in attenuation for each 10 × 10-pixel window. The slopes of lower-dose images were used as a ‘goal slope’ to create dose reductions from the original-dose images per tube voltage.

The ‘goal slope’ derived from the phantom images was used in a custom Python (version 3.7) script to introduce generated noise on images (hereafter referred to as InGen). InGen implements three steps to add noise to existing X-ray images, such as those available in MIMIC.

First, the original-dose full-resolution images were divided into twenty equal-sized grey value threshold windows (e.g., 0/20th–1/20th, 1/20th–2/20th, etc.). These twenty thresholds were determined during a preliminary phase and based on the visual inspection of simulations with various numbers of thresholds. For each window, the mean grey value was multiplied by the ‘goal slope’ related to the intended low dose. This outcome was multiplied by an array of randomly generated values between 0.0 and 1.0, taking the same size as the image. Combining these multiplications resulted in a noise mask.

Second, the resultant noise mask was further adjusted to increase visual agreement with real noise by using a multiplication factor of 6 or 9 to adjust the noise level to the ‘goal noise level’ of the phantom images. These multiplication factors were determined during the preliminary visual inspection while comparing the resultant slope to the ‘goal slope’, ensuring clinical representation of the generated low-dose simulations. Further optimization was performed by applying a zero-centered Gaussian filter with unit standard deviation to soften the noise.

In the last step, the processed noise mask was added to the original image, after which a correction was applied to restore the range of grey values present in the original image. This step, based on grey value thresholding followed by histogram correction, is crucial as it ensures that the grey values in the simulated low-dose image match those of a normal exposure, which were lost by subtracting the noise mask. The result is a simulated low-dose image that replicates what was initially an image with normal exposure.

The above steps were performed using Python 3.7 in combination with Scipy (version 1.4.1) and Skimage (version 0.16.2).

### 2.2. AI Model Training

The datasets MIMIC-CXR v2.0.0, -JPG 2.0.0, and -IV v0.4 were used for training [28,29,37]. These consist of anonymized patient information (e.g., date of birth and gender), full-resolution and full bit-depth DICOM-format chest radiographs, and their fourteen corresponding diagnoses. Each of the fourteen diagnoses is expressed as a binary label, each indicating the presence or absence of the related pathology. All MIMIC data were used in accordance with the PhysioNet Credentialed Health Data Use Agreement 1.5.0.

**Figure 2:**
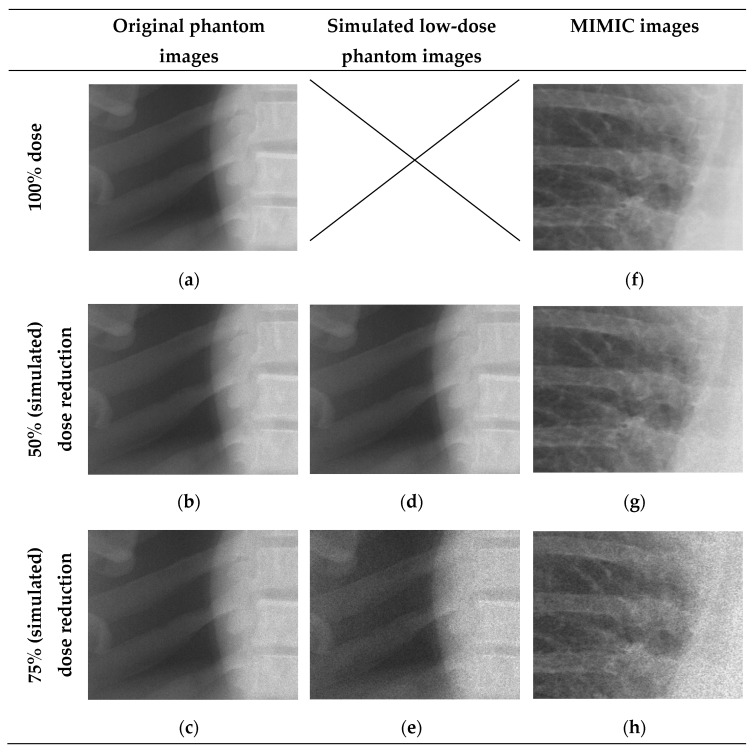
Images representative of the real and simulated low-dose images based on phantom images and MIMIC-CXR. Left: images (**a**–**c**) were acquired using an anthropomorphic phantom (2 mAs, 1.2 mAs, 0.6 mAs). Middle: images (**d**,**e**) showing 50% and 75% simulated dose reductions based on image a. Right: Images (**f**–**h**) show original MIMIC-CXR with simulated 50% and 75% dose reductions.

**Figure 3:**
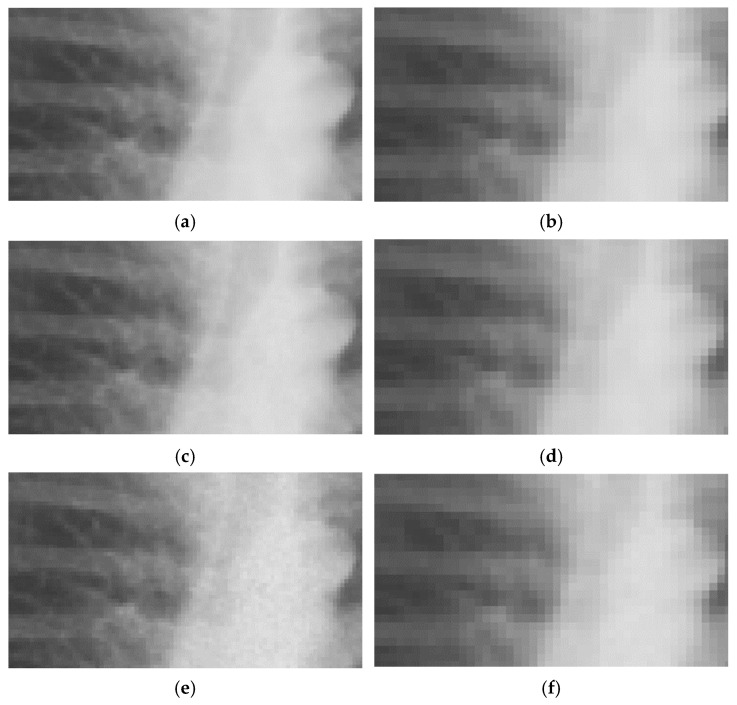
Representative examples of MIMIC-CXR images with different noise levels on training and testing resolutions. LR ((**a**–**c**): 254 × 305 px), ULR ((**d**–**f**): 127 × 152 px), and normal exposure (**a**,**d**); 50% (**b**,**e**) and 75% (**c**,**f**) simulated dose reductions.

**Figure 4:**
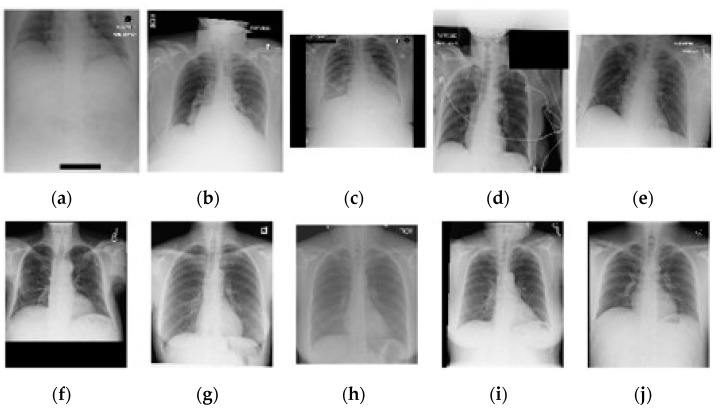
A selection of five random AP (**a**–**e**) and PA (**f**–**j**) chest images from the complete included dataset. Note the difference in image quality related to exposure, orientation, and cropping between AP and PA positioning.

The primary data source of this study is the MIMIC-CXR containing 227,835 chest radiographs. Examples of these images are shown in the Results section (see Figure 2 and Figure 3 for close-ups and Figure 4 for images of varying quality). The corresponding pathology labels for the images in MIMIC-CXR were taken from MIMIC-CXR-JPG [29,37]. Metadata from the MIMIC-CXR DICOM images were extracted using Pydicom (version 1.4.2). The relevant variables derived were tube voltage (kVp), exposure time product (mAs), dose area product (DAP, unit unknown), exposure index (EI), relative exposure index (REX) (collectively referred to as exposure parameters), and ‘acquisition date’. Both ‘anchor age’ and ‘anchor year’ from MIMIC-IV were used in combination with the ‘acquisition date’ from the DICOM metadata to derive the patient age at the time of exposure [36].

The inclusion and exclusion of pathologies and related images were performed by the first author, a radiographer with over ten years of experience, and validated using the literature [21,38,39,40,41]. Of the fourteen available pathologies, eight were included based on clinical relevance: No Finding, Fracture, Enlarged Cardiomediastinum, Cardiomegaly, Atelectasis, Edema, Pneumonia, Lung Lesion (see Table 1). Pneumothorax was excluded because of the presence of drains on 30% of 100 random pneumothorax images. Previous studies have shown that drains cause an overfit in CNN development related to pneumothorax [33,38]. Secondly, ambiguous language or limited difference in image characteristics lead to the exclusion of Lung opacity, Consolidation, Pleural other and Pleural effusion [29,33]. Lastly, images positively labelled under Support Devices were excluded from classification due to limited clinical relevancy. A radiologist with over twenty years of experience independently confirmed the choices made.

Only pathology labels with clear certainty were included (1 and 0). The uncertain label (−1), occurring in 2% of the cases was excluded, limiting the inclusion of false positives or false negatives [24,29]. The presence/absence labels were taken as true positives (1) and true negatives (0) during the training and validation of the CNNs. Additional exclusion based on image quality was not performed to circumvent an overfit on clinically unrealistic image quality.

For each of the seven pathologies, image sets were created, with 50% of the images showing the presence of the specific pathology and 50% showing its absence. Each image set was limited to one pathology label, resulting in seven separate pathology-based image sets. The maximum size per image set was 10,000 images, and each set was saved separately. The final number of images used for training, validation, and testing per pathology ranged from 4670 to 10,000, depending on the number of images available for each pathology (see Table 2). These image sets were randomly divided into training, validation, or test sets with a split ratio of 0.7, 0.2, and 0.1, ensuring independence among samples.

The training was performed on an EfficientNetB4 model pretrained with ImageNet using Keras (version 2.4.3) with a TensorFlow (version 2.4.1) backend [42]. This model was customized with a top layer (Dropout (0.2), Flatten, and Dense (1) with sigmoid activation) to facilitate binary classification per pathology.

The resolutions used were based on the approach by Sabottke et al. and available hardware resources, in which a batch of four could be used at 254 px × 305 px and a batch of eight could be used for 127 px × 152 px (respectively referred to as low resolution (‘LR’) and ultra-low resolution (‘ULR’)) [33]. A custom image data loader was used to allow for the use of 16-bit images and augmentations to simulate a broad range of geometric image acquisition variations. The implementation of image augmentation was based on the literature and was applied by Keras in its built-in augmentation during the training phase, which included a rotation ± 10 degrees, vertical flip, and fractional changes to the zoom range ± 0.01, height shift ± 0.05, width shift ± 0.1, and shear range ± 0.1 with a ‘constant’ fill mode [39,40,41,43]. Note that no augmentations were applied during testing to ensure independence of the impact of the simulated low dose on CNN performance during testing.

The values of the hyperparameters for model training were based on the literature and tuned through preliminary testing [33,39,40]. The implementation of an Adam optimizer with an initial learning rate of 1 × 10^−3^ and binary cross entropy loss function led to the optimal reduction in validation loss during these preliminary tests. Additionally, preliminary testing led to the implementation of a learning rate scheduler and an adjustment in the number of steps per epoch. The learning rate scheduler reduced the learning rate by a factor of 0.5 after each epoch, while the number of steps was reduced by a factor of 4 to increase the effect of backpropagation (therefore, each epoch was a ‘semi-epoch’). Preliminary experiments pointed towards stabilization in the training process after six epochs.

The resulting fourteen separate models, created by crossing seven pathologies with two resolutions, were trained end-to-end. Each model predicted the presence of a pathology using a range between 0 and 1. Predictions close to 0 indicated that the pathology was absent with a high certainty, while those close to 1 indicated that the pathology was present with a high certainty.

An overall investigation of the impact of simulated low-dose imaging on CNN performance was achieved by applying InGen to all images within the test image sets. Simulations were based on the ‘goal-coefficients’ and correction factors from the phantom images. Both types of parameters were based on a tube voltage of 100 kVp, taken from the MIMIC-CXR meta-data. Three separate dose levels were simulated for both LR and ULR resolutions: one for the original image dose and two for the simulated dose reductions of 50% and 75% using InGen. Please note that these simulated reductions represent images with 50% and 25% of the original dose.

### 2.3. Data Analysis

A visual face validity check was performed to investigate the clinical representation of simulated doses by using 15 randomly selected images. The corresponding author and an experienced radiologist independently performed this check. Additional image quality investigation was performed using the structural similarity index (SSIM) and peak signal to noise (PSNR). Both SSIM and PSNR were determined for the original resolution, LR and ULR [44].

The performance of the trained CNNs was operationalized using the AUROC obtained from predictions on independent test sets for each pathology, resolution, and dose level. The use of AUROC facilitates comparison with relevant literature and, due to its monotonic relationship with sensitivity and specificity, a higher AUROC indicates better clinical performance. To assess the significance of the effect of simulated dose reductions on CNN performance, the ROC curves of the original image predictions were compared to those of the simulated dose reductions using a DeLong test [45,46]. This comparison was performed for both resolutions independently, and for each of the seven pathologies.

To provide insights into potential clinically relevant outcomes, additional preliminary investigations were carried out. The differences in exposure parameters and age for PA and AP positioning were tested using a Mann-Whitney U test for continuous non-normally distributed data. Analyses were performed using Scikit-learn (version 0.22.1) to determine the AUROC and SciPy (version 1.4.1) for all other statistical analysis with a significance level of *p* < 0.05.

Finally, a cursory estimation on overall image quality related to image acquisition was performed on 100 random images for both AP and PA positioning.

## 3. Results

### 3.1. Simulated Low-Dose Radiography

A visual face validity check was performed on three low-dose simulation for five random full resolution MIMIC images. Both the first author and an experienced radiologist independently agree on the clinical representability of the low-dose simulations. Figure 2 uses representative images and shows a difference in noise levels between high and low exposure areas (e.g., lung versus bone), and a softened ‘salt and pepper’ appearance. Also note the higher degree of noise for the lower dose simulations, most notably the 75% low-dose phantom image compared to the 75% low-dose simulations. This indicates a larger than expected increase in noise.

Objective evaluation of image quality on the five full resolution images at the three noise levels, comparing the original images to the 50% and 75% low-dose simulations, resulted in an SSIM > 0.992. The PSNR for the original phantom images was 53.1 dB and 50.0 dB while the low-dose simulated full resolution images resulted in 55.7 ± 0.5 dB and 46.1 ± 0.5 dB for the 50% and 75% simulations, respectively. This shows a similar impact of increased noise on the phantom and MIMIC images verifying the face-validity.

After lowering images resolution to LR and ULR used for CNN input, the noise generated by InGen is less prominent compared to the original full-resolution images, as shown in Figure 3. Note the difference in expression of noise in the examples shown in Figure 2 and Figure 3. SSIM shows comparable results as the full resolution images with all values > 0.998. This indicates a smaller impact of noise on structural similarity. PSNR measurements were higher for lower resolutions, as shown in Table 3, which also indicates a smaller effect of noise on lower resolutions.

### 3.2. Investigation of Performance Through AUROC

#### 3.2.1. Overall Investigation

**Figure A1:**
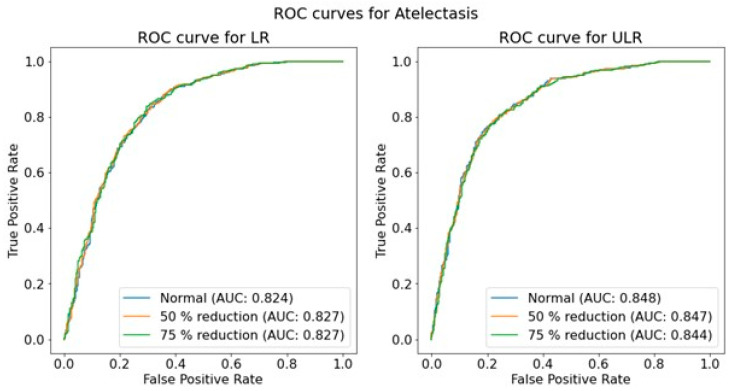
ROC for Atelectasis for low and ultra-low resolutions and all dose levels.

**Figure A2:**
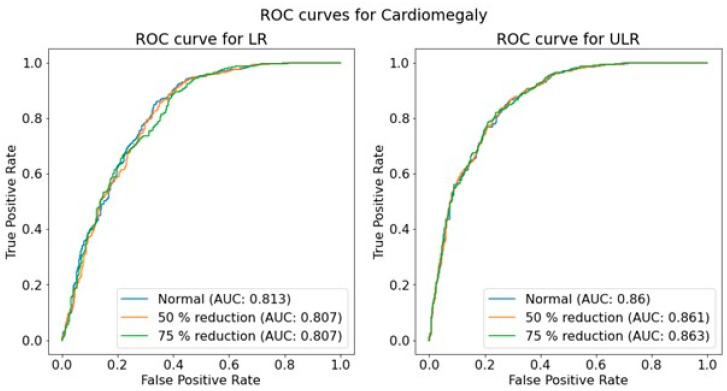
ROC for Cardiomegaly for low and ultra-low resolutions and all dose levels.

**Figure A3:**
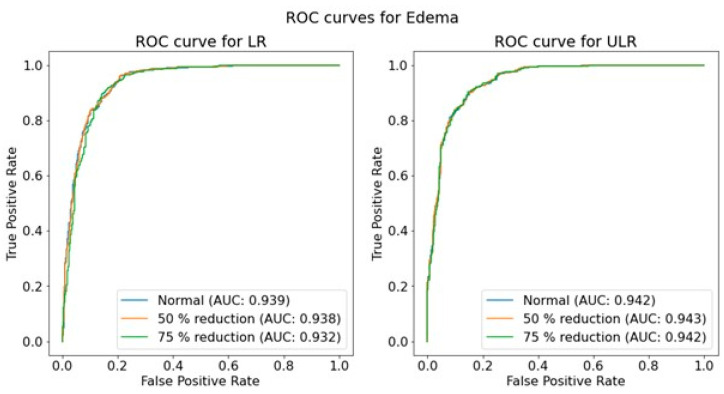
ROC for Edema for low and ultra-low resolutions and all dose levels.

**Figure A4:**
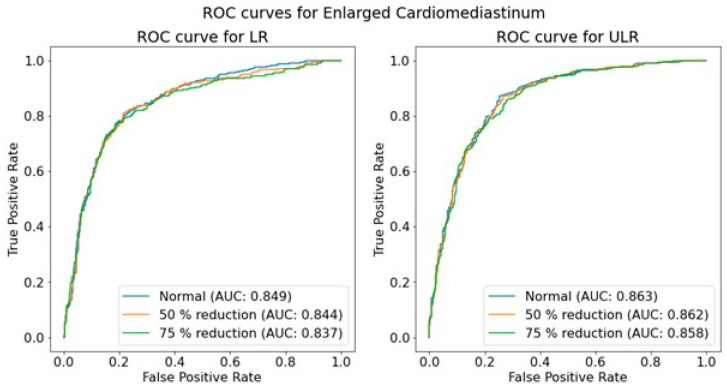
ROC for Enlarged Cardiomediastinum for low and ultra-low resolutions and all dose levels.

**Figure A5:**
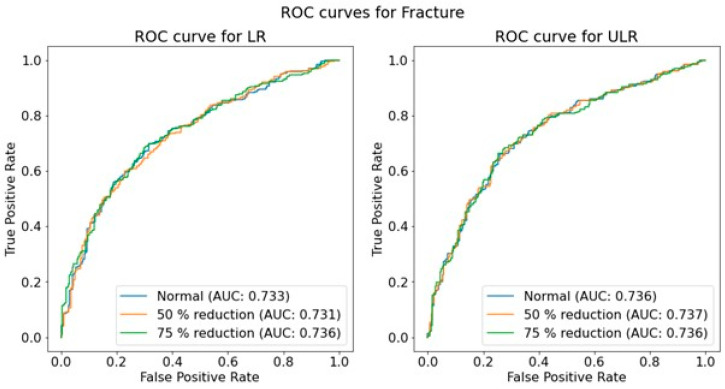
ROC for Fracture for low and ultra-low resolutions and all dose levels.

**Figure A6:**
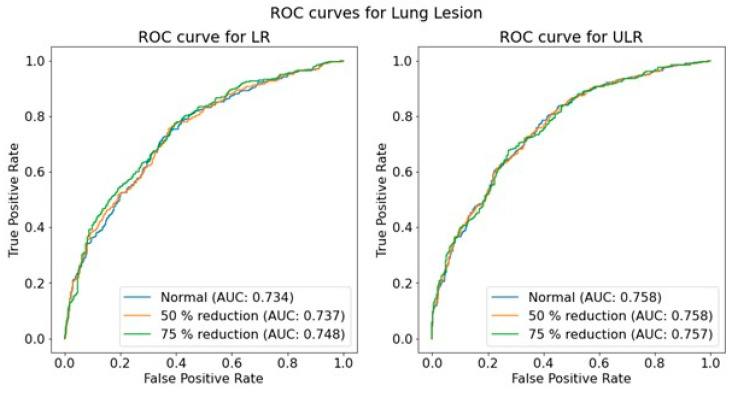
ROC for Lung Lesion for low and ultra-low resolutions and all dose levels.

**Figure A7:**
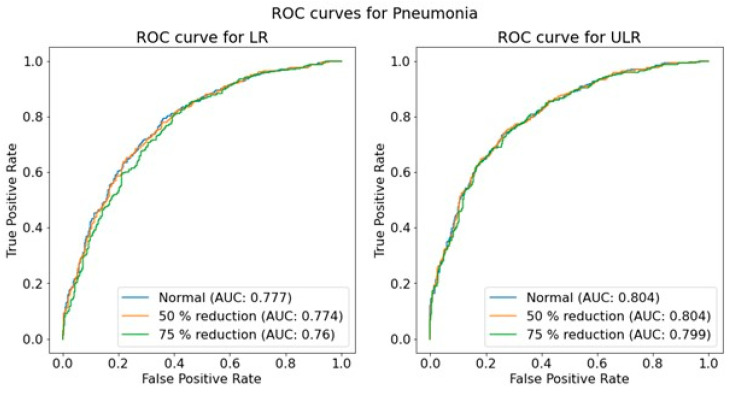
ROC for Pneumonia for low and ultra-low resolutions and all dose levels.

Simulated dose reductions resulted in minimal changes in CNN performance expressed by the AUROC for all models. The CCN models in the ULR appear more stable than the LR (see Table 4 for the AUROC values). The average absolute deviation in the AUROC for all LR predictions is 0.005 (SD = 0.004), with none exceeding the change of −0.014 for LR Pneumonia. The change in the AUROC for ULR predictions is smaller, with an absolute mean change of 0.002 (SD = 0.001) and a maximum change of −0.005 for Pneumonia. The nature of change is equal across the ROC, as shown by ROC curves presented in Figure A1, Figure A2, Figure A3, Figure A4, Figure A5, Figure A6 and Figure A7 provided in Appendix A.

Analyses of the ROCs using the DeLong test to compare the original image predictions with the low-dose simulation prediction resulted in a *p* < 0.040 in 6 out of 28 cases (see Table 4). Five of these significant differences are found within the LR predictions and one in the ULR predictions.

#### 3.2.2. Investigation of Division by Gender

The changes related to simulated low-dose imaging differ by gender; the absolute mean change in prediction for LR male images is 0.006 (SD = 0.004) and that for LR female images is 0.006 (SD = 0.005). However, the absolute changes in ULR male and female images are lower, with respective mean changes of 0.003 (SD = 0.003) and 0.002 (SD = 0.002) (Table 5). Comparisons of the original ROC to the simulated low-dose ROCs using the DeLong test show nine significant findings (Table 5).

The overall effect of the dose reductions is comparable to the overall investigation, and the difference between males and females never exceeds 0.044 for LR Lung Lesion 50%, with an absolute mean difference of 0.017 (SD = 0.011) for all AUROCs.

#### 3.2.3. Investigation of Division by View Position

The overall predictions separated for PA and AP show a comparable effect of simulated low-dose imaging on model predictions. The absolute mean change in LR PA and LR AP predictions are 0.015 (SD = 0.017) and 0.007 (SD = 0.005), respectively, while URL PA and URL AP predictions are 0.002 (SD = 0.003) and 0.003 (SD = 0.004), respectively (Table 6). The DeLong tests for comparing the original ROCs to 50% and 75% dose reductions show *p*-values < 0.049 in nine comparisons, five of which belong to LR PA. The remaining four significant differences are spread across the remaining resolutions and positioning (Table 6).

However, PA and AP positioning shows a difference for all AUROCs, with a maximum of 0.189 for LR Cardiomegaly and an absolute mean difference of 0.087 (SD = 0.061). To investigate this difference between PA and AP further, an additional analysis was performed.

Further analysis of the DAP, the EI, the REX, the mAs, and age showed a higher median and interquartile range (IRQ) for AP (see Table A1 in Appendix B). In contrast to the aforementioned variables, the tube voltage was significantly lower for AP images. An analysis of the exposure parameters and age between PA and AP using the Mann–Whitney U test resulted in *p* < 0.001 for all situations, showing a significant difference.

Supplementary information related to image quality was obtained using a cursory inspection of 100 random PA and AP images, of which some representative examples are shown in Figure 4. A deviation in average image quality related to image acquisition was visible in 15 PA cases and 52 AP cases. The largest deviations appear on AP images and are related to inspiration, field size, exclusion of anatomy, rotation (longitudinal and AP axis), and cropping. In 13 PA and 73 AP cases, lines are visible (e.g., intravenous drips, ECG, and port-a-cath).

## 4. Discussion

The aim of this study was to design a low-dose simulation and to evaluate the effect of this low-dose simulation on the CNN performance in plain chest radiography. The low-dose simulation results in clinically representative simulations; however, the visual impact of the generated noise is limited at lower resolutions often used in CNN development. The effect of the simulated low dose on performance, expressed by the AUROC, is minimal, although significant in 6 of 28 cases. These results are comparable for all included pathologies and both resolutions. These findings indicate the robustness of CNNs toward noise, making dose reductions in CNN applications for chest imaging feasible.

This study focused on developing a low-dose imaging simulation; it is important to note that the approach used approximates the noise characteristics in radiography. Clinical representativeness was improved by applying grey value thresholds and correction factors. The validation of the low-dose simulations involved two steps. First, a visual face validity check was performed at full resolution with the help of an experienced radiologist, indicating a clinically relevant simulation. Second, an objective analysis was performed using a PSNR at the original resolution to verify the visual assessment. The low-dose simulation does not account for variations in SID, specific exposure parameters, patient characteristics, and variations in equipment used for image acquisition. Due to the two-dimensional nature of the images and limited information on patient and equipment influences, individualized simulated low-dose images based on MIMIC are not feasible. The low-dose simulation in this study was based on the mean tube voltage and SID from the MIMIC data, ensuring a relevant low-dose simulation at the population level. Separate noise components (e.g., thermal and scatter noise) were also not considered. However, the noise intensity surpasses that of the individual components; so, the current approach is comparable to other approaches in the literature [34,35]. Before implementing this simulation on a lower or individual scale, care should be taken to assess the simulation’s representativeness on individual patients.

The chosen approach for low-dose simulation may not be as accurate as more complex methods [34,35]. It is important to note that the subjective and objective results show that a reduction in resolution has a large impact on the expression of noise. Any deviations caused by limitations in the low-dose simulation are therefore expected to be corrected by a reduction in resolution. This is substantiated by the DeLong tests, which show five significant differences for LR AUROCs compared to the one for the ULR in the overall analysis. Given the dependence of resolution, further investigations comparing higher and lower resolutions are needed. Nonetheless, the scientific relevancy is not to be dismissed as low- and high-resolution ensemble models could combine robustness towards the effect of lower doses with access to more information.

A benefit of the low-dose simulation compared to multiple exposures is that patient positioning did not vary between doses. The focus on the effect of simulated doses was maintained. One way to provide a more comprehensive clinical validation could be the use of human cadavers, which can be exposed repeatedly without the risks associated with radiation exposure, unlike living human subjects [47]. Although this study shows the feasibility of dose reduction in plain chest imaging when using CNNs, further investigation is required. For instance, the findings are hopeful as they indicate possibilities for modalities such as computed tomography and fluoroscopy involving higher doses.

The CNN performance encountered, with an average AUROC of 0.812 ± 0.066, for all LR and ULR models in this study is comparable to those described in other publications [24,33,38,48]. While comparable to related studies, the AUROCs are lower than the top results, which exceed 0.95 [22]. Nonetheless, the AUROC achieved in this study is encouraging considering the limited training optimization (e.g., a limited number of epochs) and the inhomogeneous nature of the data (e.g., the negative impact of positioning). While further optimization of CNN performance, such as by extending the number of training epochs, is feasible, it falls outside the scope of this study.

Combining the poor performance of AP predictions with the significantly older age of patients in this group, this study suggests that there is ageism related to the implementation of AI models. This is further substantiated by the fact that AP imaging is often the only one performed on patients who are unable to stand erect due to their ill health. This limitation often results in inferior positioning and decreased image quality. It is important for radiographers to realize this as they play a crucial role in optimizing image acquisition. Another suggestion could be to differentiate between PA and AP images when implementing CNNs for the diagnosis of chest X-rays.

## 5. Conclusions

The low-dose simulation developed during this study provides clinically representative low-dose images from original exposure images. The effect of these low-dose simulations on CNN performance is very small, although significant in 6 of 28 cases. This limited effect is likely caused by reduced noise at the low resolutions used in this study. The robustness of the models toward dose reduction suggests the potential for further dose reductions in CNN-based medical imaging applications, warranting further research.

Additionally, the current study revealed that CNN performance is lower for AP images compared to PA images, which may have a more significant impact on elderly patients. This highlights the importance of tailored image acquisition by radiographers regarding the implementation of AI in clinical practice.

## Figures and Tables

**Table 1:** Overview of included and excluded pathologies.

Included	No Finding, Fracture, Enlarged Cardiomediastinum, Cardiomegaly, Atelectasis, Edema, Pneumonia, and Lung Lesion
Excluded	Pneumothorax, Support Devices, Lung Opacity, Consolidation, Pleural Effusion, and Pleural Other

**Table 2:** Total number of images per pathology per image set for training, validation, and test sets.

Pathology	*n* Training	*n* Validation	*n* Test
Atelectasis	7000	2000	1000
Cardiomegaly	7000	2000	1000
Edema	7000	2000	1000
Enlarged Cardiomediastinum	6730	1923	961
Fracture	3269	934	467
Lung Lesion	5030	1437	718
Pneumonia	7000	2000	1000

**Table 3:** PSNR results of the comparison between the original-dose images to 50% and 75% low-dose simulations of four randomly chosen images.

	Original Resolution	LR	ULR
Simulation 50%	55.7 ± 0.5	73.8 ± 0.8	77.6 ± 1.3
Simulation 75%	46.1 ± 0.5	62.7 ± 0.8	64.9 ± 1.3

**Table 4:** AUROC for all model prediction test sets (original dose and simulated dose reductions of 50% and 75%) at different resolutions.

Pathology	LR			ULR		
	Original	50%	75%	Original	50%	75%
Atelectasis	0.824	0.827 *	0.827	0.848	0.847	0.844
Cardiomegaly	0.813	0.807	0.807	0.861	0.861	0.863
Edema	0.939	0.938	0.932	0.942	0.943	0.942
Enlarged Cardiomediastinum	0.849	0.844 *	0.837 *	0.863	0.862 *	0.858
Fracture	0.733	0.731	0.736	0.736	0.737	0.736
Lung Lesion	0.734	0.737	0.748 *	0.758	0.758	0.757
Pneumonia	0.777	0.774	0.760 *	0.804	0.804	0.799

All significant differences between the AUROCs of the original dose and simulated low dose are marked with ‘*’.

**Table 5:** AUROC for all model predictions (original dose and simulation dose reductions of 50% and 75%) at different resolutions separated by gender.

	LR					
	M			F		
Pathology	Original	50%	75%	Original	50%	75%
Atelectasis	0.833	0.835	0.837	0.818	0.821	0.821
Cardiomegaly	0.822	0.813	0.82	0.803	0.801	0.793
Edema	0.925	0.929	0.916	0.952	0.947	0.949
Enlarged Cardiomediastinum	0.86	0.856	0.85	0.835	0.828 *	0.820 *
Fracture	0.727	0.733	0.738	0.731	0.723	0.73
Lung Lesion	0.755	0.758	0.768	0.712	0.714	0.729
Pneumonia	0.759	0.759	0.752	0.791	0.786 *	0.767 *
	**ULR**					
	**M**			**F**		
**Pathology**	**Original**	**50%**	**75%**	**Original**	**50%**	**75%**
Atelectasis	0.856	0.856	0.856	0.845	0.842 *	0.839
Cardiomegaly	0.866	0.869 *	0.873 *	0.853	0.853	0.852
Edema	0.94	0.94	0.94	0.944	0.944	0.942
Enlarged Cardiomediastinum	0.874	0.872	0.863 *	0.852	0.851	0.851
Fracture	0.728	0.733 *	0.734	0.735	0.734	0.73
Lung Lesion	0.758	0.757	0.754	0.755	0.756	0.758
Pneumonia	0.799	0.799	0.793	0.809	0.808	0.804

All significant differences between the AUROCs of the original dose and simulated low dose are marked with ‘*’.

**Table 6:** AUROC for all model predictions (original dose and dose reductions of 50% and 75%) at different resolutions separated by PA and AP positioning.

	LR					
	PA			AP		
Pathology	Original	50%	75%	Original	50%	75%
Atelectasis	0.845	0.849	0.846	0.709	0.711	0.718
Cardiomegaly	0.859	0.853	0.841 *	0.681	0.664 *	0.675
Edema	0.942	0.944	0.936	0.884	0.881	0.876
Enlarged Cardiomediastinum	0.779	0.747 *	0.717 *	0.772	0.769	0.768
Fracture	0.691	0.698	0.704	0.696	0.683 *	0.696
Lung Lesion	0.755	0.758	0.779 *	0.645	0.65	0.653
Pneumonia	0.776	0.773	0.762 *	0.708	0.704	0.697
	**ULR**					
	**PA**			**AP**		
**Pathology**	**Original**	**50%**	**75%**	**Original**	**50%**	**75%**
Atelectasis	0.854	0.853	0.852	0.765	0.765	0.76
Cardiomegaly	0.9	0.901	0.902	0.755	0.756	0.759
Edema	0.957	0.956	0.953	0.887	0.884	0.887
Enlarged Cardiomediastinum	0.84	0.839	0.828 *	0.77	0.767	0.766
Fracture	0.679	0.681	0.678	0.699	0.703	0.714 *
Lung Lesion	0.786	0.786	0.787	0.67	0.67	0.671
Pneumonia	0.802	0.801	0.793	0.75	0.75	0.747

All significant differences between AUROCs of the original dose and simulated low dose are marked with ‘*’.

## Data Availability

The MIMIC data used in this study are available online; project information and access to the data are provided by MIT through https://mimic.mit.edu/docs/ (accessed on 17 March 2025). The InGen script itself is available at https://github.com/HendrikBE/LDSim/blob/main/noise_gen (accessed on 17 March 2025); data used for the calibration of InGen are also available through this repository.

## Appendix B

**Table A1:** Median and IQR of acquisition parameters and patient age for AP and PA images.

	PA			AP		
	Median	IQR	n	Median	IQR	n
Age (y)	57	27	5772	68	24	6508
DAP (unknown)	1.1	0.8	4376	2.1	1.6	4282
EI (n.a.)	157.5	47.4	5866	464.7	322.7	3898
REX (n.a.)	1321	1167	5770	1762	265	6508
Tube voltage (kVp)	110	10	5766	90	0	4682
Exposure time product (mAs)	2.5	1.5	5766	3.2	0.8	4682
